# CT-based navigation for total hip arthroplasty: a meta-analysis

**DOI:** 10.1186/s40001-023-01437-4

**Published:** 2023-10-18

**Authors:** Filippo Migliorini, Francesco Cuozzo, Francesco Oliva, Joerg Eschweiler, Frank Hildebrand, Nicola Maffulli

**Affiliations:** 1 Department of Orthopaedic, Trauma, and Reconstructive Surgery, RWTH University Hospital, Pauwelsstraße 30, 52074 Aachen, Germany; 2 Department of Medicine, Surgery and Dentistry, University of Salerno, 84081 Baronissi, SA Italy; 3 School of Pharmacy and Bioengineering, Keele University Faculty of Medicine, ST4 7QB Stoke On Trent, England; 4 Queen Mary University of London, Barts and the London School of Medicine and Dentistry, Centre for Sports and Exercise Medicine, Mile End Hospital, E1 4DG London, England; 5 Department of Orthopaedic and Trauma Surgery, Academic Hospital of Bolzano (SABES-ASDAA), Teaching Hospital of the Paracelsus Medical University, 39100 Bolzano, Italy; 6 Department of Human Sciences and Promotion of the Quality of Life, San Raffaele Roma Open University , Rome, Italy; 7 Faculty of Medicine and Psychology , Univeristy of Rome La Sapienza , Rome , Italy

**Keywords:** Hip, Arthroplasty, Navigation, CT based

## Abstract

**Introduction:**

Computer tomography (CT) based navigation is considered by some authors as an advance in total hip arthroplasty (THA). A meta-analysis was conducted to compare CT based versus conventional THA in terms of surgical duration of the procedure, leg length difference, acetabular cup position, and rate of dislocation.

**Material and methods:**

The present study was conducted according to the PRISMA 2020. In December 2022, the following databases were accessed: PubMed, Web of Science, Google Scholar, Embase with no time constrain. All the clinical studies comparing CT based navigation versus the conventional THA were accessed.

**Results:**

Data from 1801 procedures were collected. The mean age of the patients was 61.6 ± 5.3 years, and the mean BMI was 26.9 ± 2.3 kg/m^2^. There was between studies comparability at baseline in terms of age, BMI, pain score, Harris hip score, leg length discrepancy (P > 0.1). The navigated group demonstrated lower leg length discrepancy (P = 0.02), and lower degrees of cup anteversion (P = 0.002). Similarity was found in cup inclination (P = 0.98), surgical duration (P = 0.3), and the rate of dislocation (P = 0.6).

**Conclusion:**

CT guided THA may have the potential to increase the accuracy of acetabular positioning and reduce the leg length discrepancy. Current evidence is very limited and heterogeneous, and no recommendations can be inferred. Further investigations are required to definitely clarify the role of CT based THA in current practice.

## Introduction

Total hip arthroplasty (THA) restores joint function and patient quality of life [1, 2]. Proper implant positioning is necessary to achieve long term THA survivorship. Dislocation, impingement, pelvic osteolysis, acetabular migration, and inlay erosion are common following acetabular components malposition [3, 4]. A 45° ± 10° of cup inclination and a 15° ± 10° of cup anteversion are recommended [5–7]. In patients with leg length discrepancy, back pain, gait impairment, greater rate of aseptic loosening, and dissatisfaction are common [8–11].

Computer tomography (CT) based THA uses algorithms and tracking systems to detect anatomical features, limb axes, and joint orientation to assist surgeons [12, 13]. Several studies compared CT based THA versus the conventional freehand procedure [13–18]. Comparative studies were however not conclusive, and the application of CT based THA is still controversial [19–23]. Recently published evidence which has not yet been included in a systematic review evidenced that the CT based THA is a valuable option to perform total hip arthroplasty, presenting some advantages over the classical freehand technique [24]. A meta-analysis was conducted to compare surgical duration, leg length discrepancy**,** cup anteversion and inclination, and rate of dislocation between these two different modalities.

## Material and methods

### Eligibility criteria

All the clinical investigation comparing CT based navigation versus the conventional freehand THA were accessed. Level I to III of evidence, according to Oxford Centre of Evidence-Based Medicine [25], were eligible. Animals, in vitro, biomechanics, computational, and cadaveric studies were not eligible. Given the authors language capabilities, articles in English, German, Italian, French and Spanish were eligible. Reviews, opinions, letters, editorials were not considered. Only studies published in peer reviewed journals were considered. Studies which used innovative implants, materials, or experimental rehabilitation programs were not considered. Only studies which report the outcomes of CT based navigation and quantitative data under the outcomes of interest were suitable. Other types of navigation methods (e.g. imageless) were not eligible.

### Search strategy

This systematic review followed the Preferred Reporting Items for Systematic Reviews and Meta-Analyses: the 2020 PRISMA statement [26]. The PICO algorithm was stated:

- P (Population): end stage hip osteoarthritis;
- I (Intervention): CT-based navigation THA;
- C (Comparison): conventional freehand THA;
- O (Outcomes): radiological parameters, surgical duration, dislocations.

In December 2022, the following databases were accessed: PubMed, Web of Science, Google Scholar, Embase with no time constrains. The keywords used for the search were: *hip, total hip arthroplasty, replacement, prosthesis, osteoarthritis, anteversion, inclination, lower limb, leg discrepancy, radiological, complications, dislocation.*

### Selection and data collection

The literature search was conducted by two authors (**;**) independently. Titles and abstract of interest were screened and the full-text of the articles of interest were accessed. If the full-text was not accessible, the article was excluded from the present investigation. The bibliography of the full-text articles were also screened for inclusion. Disagreements were debated, and the final decision was taken by a third author (**).

### Data items

Two authors (**;**) independently performed data extraction. The following data were extracted: author and year, journal, study design, number of procedures, sex of the patients, mean age at operation, type of intervention and surgical approach, type of navigation system. The following data were retrieved at last follow-up: mean cup inclination and anteversion, surgical duration, leg length discrepancy, rate of dislocations.

### Methodological quality assessment

The methodological quality assessment was performed using the Review Manager software version 5.3 (The Nordic Cochrane Collaboration, Copenhagen). Two authors (**;**) evaluated the risk of bias of each included study using the Cochrane risk of bias tool. The following biases were evaluated: selection, detection, attrition, reporting, other source of biases. Disagreements were debated, and the final decision was taken by a third author (**). To assess the overall risk of publication bias, the funnel plot was evaluated. Asymmetries of the plot indicate higher risk of bias.

### Synthesis methods

The statistical analyses were performed by the main author (**). For descriptive statistics, the IBM SPSS version 25 was used. The Shapiro–Wilk test has been performed to investigate data distribution. For parametric data, mean and standard deviation were evaluated. For non-parametric data, median and interquartile were evaluated. Mean difference (MD) effect measure was adopted to assess baseline comparability. Student T-test and Mann–Whitney U-test were performed for parametric and non-parametric data, with P values > 0.1 considered satisfactory. For the meta-analyses, the Review Manager 5.3 software (The Nordic Cochrane Collaboration, Copenhagen) was used. For continuous data, the inverse variance with MD effect measure was adopted, while the Mantel–Haenszel method with odd ratio (OR) effect measure was used for dichotomic data. Heterogeneity was investigated using the Higgins I^2^ and $\chi$ ^2^ tests. If $\chi$ ^2^ < 0.05 and I^2^ > 75%, high heterogeneity was found. A fixed method effect model was used as default; if high heterogeneity was found, a random effect model was used. The confidence interval (CI) was set at 95% in all comparisons. Overall P values of < 0.05 were considered statistically significant.

## Results

### Study selection

**Fig. 1:**
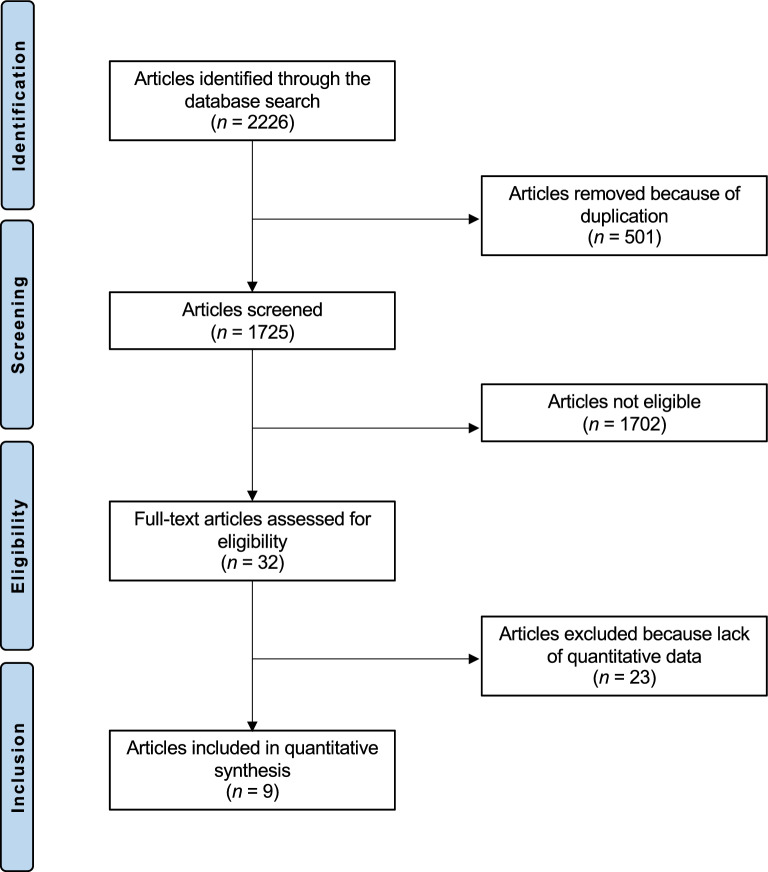
Flow chart of the literature search

The literature search identified 2226 articles related to navigated arthroplasty. Of them, 501 were duplicates. Further 1702 articles were excluded: did not focused on CT based (N = 1395), not focused on hip (N = 177), study type (N = 103), other (27). Further 23 studies were excluded as did not report quantitative data under the outcomes of interest. This left 9 articles for inclusion: 3 randomized clinical trials (RCTs), 2 prospective, and 4 retrospective clinical studies (Fig. 1).

### Methodological quality assessment

**Fig. 2:**
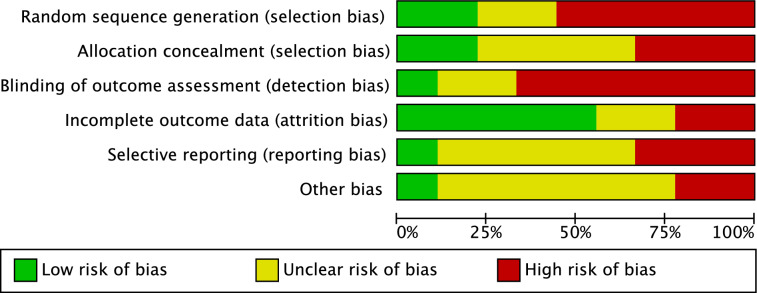
Methodological quality assessment

Given the high ratio of non randomised studies (6 of 9 studies), the risk of selection bias was moderate to high. Moreover, 67% (4 of 6) of the included studies had a retrospective design, which further increase the risk of selection bias by allocation concealment. Given the lack of blinding in most studies, the risk of detection bias was moderate-high. Attrition bias and reporting biases were both moderate-low, and the risk of other biases was moderate. Concluding, the methodological assessment demonstrated a moderate risk of bias (Fig. 2).

### Risk of publication bias

**Fig. 3:**
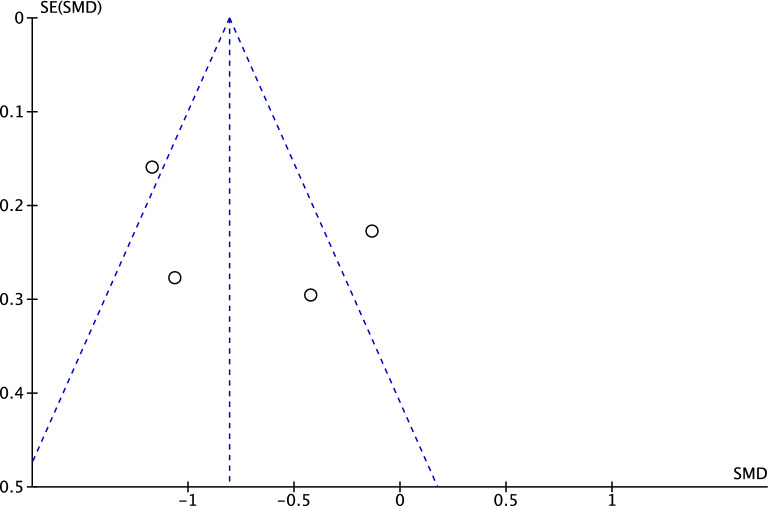
Funnel plot

The funnel plot of the most reported outcome (cup anteversion) has been performed to evaluate the risk of publication bias. The plot demonstrated high symmetry, indicating a very low risk of publication bias (Fig. 3).

### Study characteristics and results of individual studies

Data from 1801 procedures were collected, 58% in women. The mean age was 61.6 ± 5.3 years. The mean BMI was 26.9 ± 2.3 kg/m^2^.There was between group comparability at baseline in terms of age, BMI, VAS, Harris hip score, leg length discrepancy (P > 0.1). Generalities and patient baseline characteristics of the included studies is shown in greater detail in Table 1.

**Table 1:** Generalities and patient baseline of the included studies (RCT: randomised clinical trial)

Author, year	Journal	Design	Procedures (n)	Female	Mean age	Intervention	Approach	Navigation system
Confalonieri et al. 2008 [27]	*Orthopedics*	Retrospective	22	59%	60.4	Image Navigated	Posterolateral	Orthopilot
			22	59%	60.8	Conventional	Posterolateral	
Domb et al. 2015 [18]	*Arthroplasty*	Retrospective	43		64.7	Image Navigated	Anterior	Vectorvision 3.0
			708		64.7	Conventional	Posterior	
Haaker et al. 2007 [16]	*Arthroplasty*	Retrospective	98	64%	66.9	Image Navigated	Anterolateral	Optotrak Surigate
			69	62%	63.4	Conventional	Anterolateral	
Kalteis et al. 2006 [24]	*Bone Joint J*	RCT	30	40%	63.9	Image Navigated	Transgluteal	Vectorvision BrainLab 3.0
			30	57%	64.7	Conventional	Transgluteal	
Leenders et al. 2002 [28]	*Wilet Interscience*	RCT	50	58%	61	Image Navigated	Anterolateral	Surgi-Gate system
			50	58%	64.9	Conventional	Anterolateral	
Lin et al. 2011 [29]	*Arthroplasty*	Prospective	22			Image Navigated	Posterior	Stryker CT-Hip System
			25	40%	63.5	Conventional	Posterior	
Murphy et al. 2006 [17]	*J Orthop Rel Res*	Prospective	185	47%	56.1	Image Navigated	Transgluteal	
			189	50%	50.4	Conventional	Transgluteal	
Sugano et al. 2012 [30]	*Clin Orthop Rel Res*	Retrospective	60	88%	53	Image Navigated	Posterolateral	Optotrak UltraSPARC
			120	83%	53	Conventional	Posterolateral	
Verdier et al. 2016 [31]	*Orthop Traumatol*	RCT	39	54%	67	Image Navigated	Anterolateral	Orthopilot
			39	54%	68	Conventional	Anterolateral	

### Results of syntheses

**Fig. 4:**
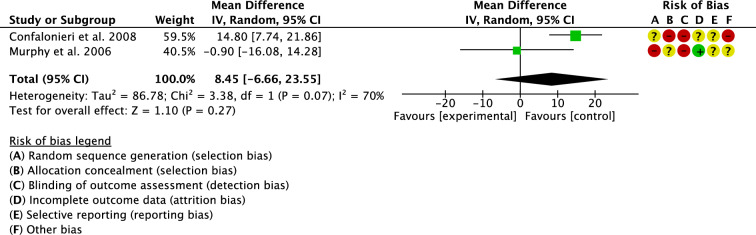
Meta-analysis of the comparison: surgical duration

Two studies investigated surgical duration [17, 27]. The overall effect resulted statistically not significant (P = 0.3), evidencing similarity between the groups (Fig. 4).

**Fig. 5:**
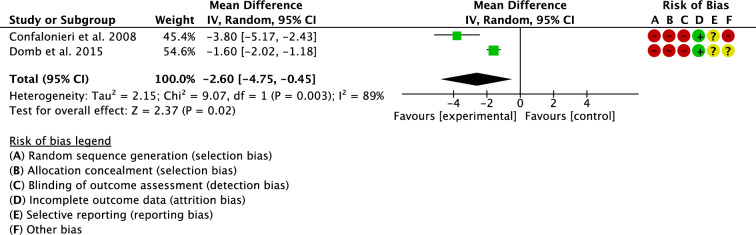
Meta-analysis of the comparison: leg length discrepancy

Two studies investigated leg length discrepancy [18, 27]. The navigated group demonstrated lower leg length discrepancy (MD -2.60; 95% CI − 4.75 to − 0.75; P = 0.02; Fig. 5).

**Fig. 6:**
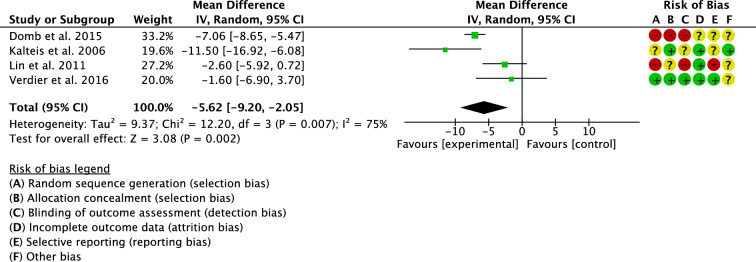
Meta-analysis of the comparison: cup anteversion

Four studies compared cup anteversion [18, 24, 29, 31]. The navigated group demonstrated lower degrees of cup anteversion (MD − 5.62; 95% CI − 9.20 to − 1.05; P = 0.002; Fig. 6).

**Fig. 7:**
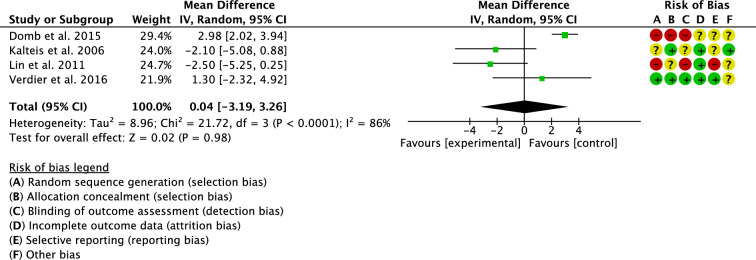
Meta-analysis of the comparison: cup inclination

Four studies compared cup inclination [18, 24, 29, 31]. The overall effect resulted statistically not significant (P = 0.98), evidencing similarity between the groups (Fig. 7).

**Fig. 8:**
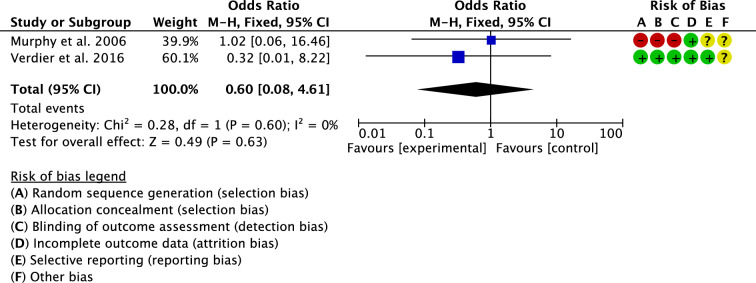
Meta-analysis of the comparison: rate of dislocation

Two studies compared the rate of dislocation [17, 31]. The overall effect resulted statistically not significant (P = 0.6), evidencing similarity between the groups (Fig. 8).

## Discussion

According to the main findings of the present study, the current evidence demonstrated that CT based THA may promote more accurate cup anteversion and lower leg length discrepancy compared to the conventional procedure. No difference was found in cup inclination, duration of surgery, and rate of dislocation between the two techniques.

CT based THA navigation resulted in similar inclination compared to the conventional procedure. However, navigated THA demonstrated a more accurate cup anteversion compared to the conventional procedure. Domb et al. [18] reported that cups in navigated THA were placed in the safe zone [32] in 100% of patients, compared to 80% in the freehand THA [18]. Kalteis et al. [24] found that 14 of 30 cups were placed in the safe zone following freehand THA, and 25 of 30 of the navigated were optimally placed. On the contrary, Lin et al. [29] evidenced similar positioning between freehand and CT based THA groups: inclination was optimal in 23 of 25 freehand THA and 25 of 25 CT navigated THA; cup anteversion was optimal in 19 of 25 conventional THA and 21 of 22 CT based THA [29]. The authors reported CT navigated THA is associated to more precise placement of the acetabular component, with low rates of malposition [29]. Verdier et al. [31] found that patients who target the optimal anteversion range was reached in 28 of 39 patients in the navigated THA, and in 17/39 of patients who underwent the freehand technique [31]. CT guided demonstrated lower leg length discrepancy compared to the conventional procedure [18, 27]. Confalonieri et al. [27] evidenced no difference between the two groups in pre-operative leg length discrepancy. Postoperatively, there were 0.4 cm of discrepancy in CT guided THA group and 0.8 cm in THA freehand group [27]. No post-operative leg length discrepancy greater than 1 cm was reported in any patient of the CT guided group [27]. Domb et at. [18] found a leg length discrepancy greater than 1 cm in 3% of 708 patients who had undergone conventional THA, and none of the navigated group (N = 43) demonstrated a discrepancy greater than 1 cm. Surgical duration was similar between the two procedures. Two studies investigated the surgical duration [17, 27]. Confalonieri et al. [27], in a population of 44 patients, showed a statistically longer surgical time with a mean time of 102.6 min in the navigated group compared to 87.7 min of free hand group. Murphy et al. [17], using specific instruments for CT based THA, reported similarity between the procedures: 177 min in the navigated group compared to the 178 min in the conventional group. This study demonstrated that the rate of dislocation between the two techniques was similar. Murphy et al. [17] found no dislocation in 185 patients treated with CT based THA, and two dislocations on 189 patients were reported in the cohort of freehand THA. Verdier et al. [31] found one dislocation in the freehand group (in a female patient with excessive anteversion) and none in the navigated group [31]. The present study indicated that CT guided THA may have the potential to increase the accuracy of acetabular positioning and reduce leg length discrepancy. To better identify the advantages of CT guided THA, high quality studies involving large cohort of patients. The current evidence is very limited and heterogeneous; therefore, no strong recommendations can be inferred. Further investigations are required to clarify the role of CT based THA.

The present study has several limitations. The limited number of clinical studies and procedures included for analysis represent the most important limitations, together with, the retrospective design of most studies. Given the limited data available in the current literature, surgical approach and/or the type of implant used were not analyzed. The description of the surgical technique was adequate in most studies. The eligibility criteria were barely reported, and often biased. Given the limited data available, perioperative data (e.g. mean blood loss, transfusion units), joint function, and patients quality of life was not possible to compare. Finally, most analyses were conducted using a random effect model, as the level of between studies heterogeneity was high. Given these limitations, results from the present study must be interpreted with caution.

## Conclusion

CT guided THA may have the potential to increase the accuracy of acetabular positioning and reduce the leg length discrepancy. The current evidence is very limited and heterogeneous; therefore, no strong recommendations can be inferred. Further investigations are required to definitely clarify the role of CT based THA in current practice, as it is unclear whether these reported differences, though of statistical significance translate in clinical relevance in terms of better function and/ or greater longevity of the implants used for THA.

## Data Availability

The datasets generated during and/or analysed during the current study are available throughout the manuscript.

## Abbreviations

THA: Total hip arthroplasty
CT: Computer tomography
MD: Mean difference
OR: Odd ratio
CI: Confidence interval
RCTs: Randomized clinical trials
